# Vaccination for Disease Prevention and Control: the Necessity of Renewed Emphasis and New Approaches

**DOI:** 10.17653/2374-9105.SSe0001

**Published:** 2014-07-09

**Authors:** Kwang Poo Chang

**Affiliations:** Department of Microbiology/Immunology Chicago Medical School/Rosalind Franklin University of Medicine and Science, North Chicago, USA

## Introduction

Vaccination was discovered initially as an effective measure to prevent infectious diseases by Edward Jenner, Louis Pasteur and other pioneers several hundred years ago.^[1]^ Since then, efforts to develop vaccines have been rather sporadic and limited, making prophylactic vaccination available only for a limited number of infectious diseases. Prophylactic vaccination has a proven record of success in childhood immunization programs worldwide, indicative of the feasibility and necessity of developing additional prophylactic vaccines. This is not just because of their unavailability for the majority of extent infectious diseases caused by known pathogenic species of viruses, bacteria, protists, fungi and helminthes, but also for the emergence of new pathogens that is estimated to occur at a rate of 3 per year.^[2]^ Serious investment in vaccine development becomes all the more urgent considering the tenacity of infectious diseases in several ways. First, most of them are impossible to eradicate, since they are zoonotic diseases, which circulate often via vector transmission among domestic and wild animals.^[3]^ Secondly, our reliance on treatment, e.g. chemotherapy over prevention for disease management has become increasingly untenable, resulting from the development of drug-resistance by the pathogens. Finally, lasting cure of infectious diseases has long been considered to require post-therapeutic development of effective immunity, since no drug used for chemotherapy is expected to reach all the intended targets, regardless of its dosages and frequencies of applications. A good example for this is perhaps the “Mississippi baby” who turned HIV-positive two year after what was thought to be the first case cured by anti-retroviral therapy when applied to the newborn.^[4]^ Taken together, there is clearly a need of our renewed emphasis on immuno-prophylaxis of all diseases by vaccination.

The agency of diseases is certainly far beyond pathogenic infections, including, for example, self-generated malignancy, metabolic disorders, cardiovascular failures and neurodegenerative diseases. While vaccines were developed originally for preventing infectious diseases, they have been also deployed to treat aforementioned non-infectious diseases based on the immunological principles derived from the knowledge of infection and immunity. Significant efforts have been devoted to the immunotherapy of cancers by vaccination to elicit neoantigen-specific cell-mediated immunity, e.g. Provenge (sipuleucel-T), the first FDA-approved vaccine for treating malignancy, i. e. prostate cancer. Other examples include the elicitation of specific antibodies for treatment of addictions by using anti-nicotine vaccines (NicVAX and TA-NIC) for smoking cessation and anti-cocaine vaccines (TA-CD) to facilitate the rehabilitation of substance abusers.^[5]^ Another vaccine elicits specific antibodies for treating Type 2 diabetes mellitus with some measure of success in the animal model tested.^[6]^ Vaccines based on similar principles are also under development for treating transient ischemic attack, stroke, epilepsy and obesity. The market demands for the prevention and treatment of these significant diseases have undoubtedly driven the commercial interests in research to develop such vaccines.

The vaccines in use today for prophylaxis of infectious diseases have been developed over many years and consist of different formats, i. e. attenuated, inactivated or killed whole pathogens, their components or toxoids. The vaccines developed recently or under development are largely recombinant products of antigenic peptides (and their cDNAs). There are on-going efforts to cost-effectively produce the peptide vaccines of good quality and quantity by using different expression systems, ranging from bacteria to yeasts to other eukaryotic cells of plant and animal origin. Immunogenicity of peptide vaccines requires co-injection with effective adjuvants. This is an area of active research with high priority, since very few of them are available for use in human immunization. Vaccination of humans with peptide vaccines plus adjuvants elicits mainly humeral antibody immunity instead of the lasting cell-mediated immunity. To elicit the latter is the major clinical approach to anti-cancer immunotherapy by individualized *exo vivo* vaccination using patients’ dendritic cells. The peptide recombinant technology is most advanced and will continue to play a dominant role in the field of vaccinology. Attenuated/inactivated whole pathogens are however well-known to be potent vaccines, which require no adjuvants to elicit lasting immunity. The classical examples for this include MMR (mumps, measles, rubella) and MMRV (MMR+chicken pox), which consist of attenuated viral pathogens, providing a life-long protection for 95% of the vaccinated population after a single immunization.^[7]^ The peptide vaccines, such as Gardasil and Cervarix for cervical cancers and other genital infections are effective, but require multiple immunizations over an extended period at a considerable cost per dose.^[8]^ Recent development of anti-malarial vaccines provides another example to illustrate the same point: The peptide vaccine, Mosquirix^[9]^ is not as effective as attenuated sporozoites of the malaria parasites.^[10,11]^ Vaccines have yet to be developed in any effective and affordable format for prophylaxis and therapy against most human and animal diseases, especially those, which plague bulk of the human population in the developing and under-developed world.

It is crucial to break through the scientific and regulatory constraints by considering alternative and novel approaches to vaccination. One such approach is to develop universal carriers for appropriate delivery of vaccines to enhance their efficacy and applicability. A testament to the appeal of this approach is the proliferation of literature in this area of research. Vaccine carriers under development include various infective agents, e.g. viruses/virosomes, bacteria, including BCG, *Salmonella, Lactobacillus and Listeria,*^[12–15]^ inert particles, e.g. liposomes,^[16]^ colloidal polymers and nano-particles.^[17–19]^ The biotechnologies for using these materials as safe and effective vaccine carriers have been under study for some time. Still ongoing is the new strategy to develop *Leishmania* as the first eukaryotic vehicle for vaccine delivery on the basis of their unique properties, e. g. the efficacy of protecting vaccine payload and homing to antigen-presenting cells and their safety by photodynamic inactivation with reactive oxygen species.^[20–23]^

## Conclusion

Clearly, disease prevention by vaccination is recognized by all as a superior strategy to manage diseases. There is an urgent need to emphasize this approach by providing incentives to continue the well-established methodologies and, in addition, to promote fresh ideas in a new direction beyond the established framework.
